# Flipping the switch: How cysteine oxidation directs tau amyloid conformations

**DOI:** 10.1016/j.jbc.2021.101309

**Published:** 2021-10-15

**Authors:** Danny M. Hatters

**Affiliations:** Department of Biochemistry and Pharmacology and Bio21 Molecular Science and Biotechnology Institute, The University of Melbourne, Melbourne, Victoria, Australia

**Keywords:** protein aggregation, prion strains, tauopathy, protein biochemistry, tau, fibril assembly

## Abstract

Tau can adopt distinct fibril conformations in different human neurodegenerative diseases, which may invoke distinct pathological mechanisms. In a recent issue, Weismiller *et al.* showed that intramolecular disulfide links between cys291 and cys322 for a specific tau isoform containing four microtubule-binding repeats direct the formation of a structurally distinct amyloid polymorph. These findings have implications in how oxidative stress can flip switches of tau polymorphism in these diseases.

The protein tau features prominently in a number of neurodegenerative diseases through its aggregation into deposits. It has normal functions in microtubule binding and signaling (1). Many mutational or posttranslational modifications, including tauopathy disease-causing mutations, are known to modulate tau aggregation by altering splicing patterns, increasing the extent of its phosphorylation, reducing its binding to microtubules, or enhancing its intrinsic aggregation propensity.

One of the intriguing features of tau aggregates is that they can act like prions in propagating new deposits (2). Prions are proteins in a particular conformation, such as an amyloid, which can self-propagate that conformation by templating the recruitment to the polymer of naïve monomers of a different conformation. One example is the prion conformation of the human prion protein, known as Scrapie, which is the cause of Creutzfeldt–Jakob disease. In yeast, rather than being pathological, prions can confer new functional capacity by regulating the activity of the protein involved in prion conformational conversion. Tau has also been observed to display features akin to prion behavior and indeed can adopt multiple different conformations (strains) that manifest differently in different neurodegenerative diseases (2). For example, cryo-EM structures of tau fibrils extracted from brain tissue of deceased patients revealed different molecular fibril structures associated with different diseases, *e.g.*, corticobasal degeneration (CBD, a form of neurodegenerative tauopathy) (3), Alzheimer’s disease (4), Pick’s disease (5), and chronic traumatic encephalopathy (6). The formation of distinct strains raises the prospect that these strains can modulate different disease phenotypes and pathomechanisms. However, the details of how strains exert such effects remain to be fully determined.

In their work, Weismiller *et al.* (7) examine the effect of cysteine oxidation state on fibril assembly. The largest isoform of tau, which was the focus of this study, contains four microtubule-binding domain repeats (htau40) and is 441 amino acids in length. Htau40 contains two cysteines at positions 291 and 322 in the second microtubule-binding repeat. Prior studies have suggested that these cysteines play critical roles in modulating fibril assembly and in stabilizing monomer and dimer conformations (8, 9); however, there was conflicting data as to whether intramolecular disulfide linkage facilitated or hindered the aggregation of tau monomers or dimers. Weismiller *et al.* sought to address the specific question of how intramolecular disulfide bonds within tau affected the aggregation of the full length htau40 isoform.

Using purified htau40 treated with hydrogen peroxide, the authors generated monomers containing intramolecular disulfide linkages. The extent of disulfide formation was shown *via* both selective labeling of any remaining free cysteines with a thiol-reactive paramagnetic reagent, which enabled detection by electron paramagnetic resonance spectroscopy, and by reduction back to free thiols, to examine the possibility of overoxidation products being formed, which are not reversible. The treatments ascertained near-complete purity for protein containing the disulfide-linked monomers and the fully reduced counterpart for comparison studies.

Through a series of *in vitro* assays geared to follow the kinetics of aggregation, the authors observed that oxidized monomers of htau40 appeared to be more compact in conformation than the reduced form of htau40 and were also shown to form fibrils capable of self-seeding. The resultant fibrils were shown to contain only intramolecular disulfide bonds, indicating there was no intermolecular disulfide shuffling occurring. In a striking result, mutants that removed the cysteines to mimic the reduced form, as well as the actual reduced forms of the wild-type htau40, were unable to be recruited to fibril seeds formed by oxidized htau40, even though these forms could be recruited to fibrils formed by reduced htau40. The opposite results also held true. These findings indicate that the fibrils formed by oxidized monomeric htau4 were structurally distinct to those formed by the reduced tau monomer, and they were unable to cross-seed each other or be recruited into the other amyloid polymorph (Fig. 1). Furthermore, addition of reducing agents to preformed fibrils made by oxidized monomers resulted in disaggregation back to the reduced monomers. This indicated that the reduced htau40 monomer is incompatible with the amyloid structure formed by the disulfide-linked monomers.

**Figure 1:**
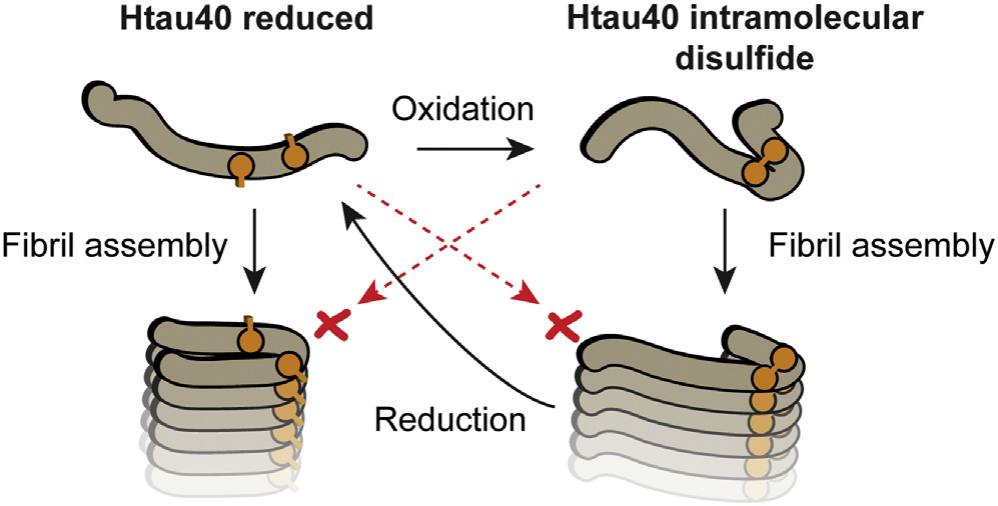
**Schematic of the two alternate pathways described in Weismiller *et al.* for how the intramolecular disulfide link switches assembly into two distinct amyloid fibrils.** The fibrils formed by the disulfide-linked monomer cannot cross-seed with the reduced htau40 and are unstable when the disulfide is reduced and dissolve back to reduced monomers. Cysteines are depicted in *orange*. The structures of the fibrils are solely indicative of different conformations between the oxidative and reduced htau40 proteins and are not meant to imply specific structural features.

This study adds a new piece to the puzzle toward understanding the structural assembly states mediating different prion structures and polymorphs. In particular, it shows that disulfide formation serves as a binary switch distinguishing two structurally different amyloid structures that are mutually incompatible in both recruitment of monomers and in cross-seeding.

This mechanism has implications for disease at two levels: one is in providing new information to a fundamental assembly mechanism accessible to the tau protein. The second is in the potential of oxidative stress, which is a marker of many if not all neurodegenerative diseases, to modulate the formation of different tau amyloid conformers and potentially to the genesis of different prion seeds. Indeed, while oxidative stress appears early in the pathological development of tauopathies, the precise role of oxidative stress remains opaque and is likely to involve many different biological processes that are interconnected. What is clear is that oxidative stress can promote the formation of phosphorylated tau as well as its aggregation (10). While the increased aggregation has been previously suggested to relate to the phosphorylation status of tau (10), the findings of Weismiller *et al.* suggest that oxidation could also flip a switch for the type of fibril polymorph that tau adopts and thereby set off different pathways of pathological events.

## Conflict of interest

The author declares that they have no conflicts of interest with the contents of this article.
